# Aberrant Expression of Cardiac Troponin-T in Lung Cancer Tissues in Association With Pathological Severity

**DOI:** 10.3389/fcvm.2022.833649

**Published:** 2022-04-11

**Authors:** Toshihiro Tsuruda, Yuichiro Sato, Masaki Tomita, Hiroyuki Tanaka, Kinta Hatakeyama, Misa Otsu, Aya Kawano, Keiko Nagatomo, Naoki Yoshikawa, Ryuji Ikeda, Yujiro Asada, Koichi Kaikita

**Affiliations:** ^1^Division of Cardiovascular Medicine and Nephrology, Department of Internal Medicine, Faculty of Medicine, University of Miyazaki, Miyazaki, Japan; ^2^Department of Diagnostic Pathology, Faculty of Medicine, Miyazaki University Hospital, Miyazaki, Japan; ^3^Department of Thoracic and Breast Surgery, Faculty of Medicine, University of Miyazaki, Miyazaki, Japan; ^4^Department of Pathology, Section of Oncopathology and Regenerative Biology, Faculty of Medicine, University of Miyazaki, Miyazaki, Japan; ^5^Department of Pathology, National Cerebral and Cardiovascular Center, Osaka, Japan; ^6^Department of Pharmacy, Faculty of Medicine, Miyazaki University Hospital, Miyazaki, Japan; ^7^Department of Pathology, Faculty of Medicine, Miyazaki University Hospital, Miyazaki, Japan

**Keywords:** lung cancer, pathology, immune checkpoint inhibitor, troponin, myocarditis

## Abstract

**Background:**

Cardiac troponin-T (*TNNT2*) is exclusively present in cardiac muscle. Measurement of *TNNT2* is used for diagnosing acute coronary syndrome. However, its expression may not be limited in myocardium. This study aimed at evaluating the expression of *TNNT2* in neoplastic tissues.

**Methods and Results:**

We used paraffin-embedded blocks of 68 patients with lung cancer (age, 68 ± 11 years old; early-stage, 33; advance-stage, 35) at Miyazaki University Hospital, Japan between January 1, 2017, and March 31, 2019. We stained the slide sections with primary monoclonal antibody against *TNNT2* protein, and assessed the frequency of positive staining, and its association with pathological severity. In addition, we examined whether *TNNT2* gene is detected in lung cancer tissues of four patients using reverse transcription-polymerase chain reaction. Immunoreactivity for *TNNT2* protein was present in the cytoplasm and nucleus of lung cancer cells. The frequency was 37% (25 of 68) in all patients and was irrespective of histologic type (six of 13, squamous cell carcinoma; 18 of 50, adenocarcinoma; 0 of 4, neuroendocrine cell carcinoma; 1 of 1, large cell carcinoma). The prevalence increased with pathological staging [9% (3 of 33) at early-stage (Stage 0–I); 63% (22 of 35) at advance-stage (Stage II–IV and recurrence)]. In addition, frequency of positive staining for *TNNT2* increased with pleural (χ^2^ = 5.877, *P* = 0.015) and vascular (χ^2^ = 2.449, *P* = 0.118) invasions but decreased with lymphatic invasion (χ^2^ = 3.288, *P* = 0.070) in specimens performed surgical resection. Furthermore, *TNNT2* mRNA was detected in the resected squamous cell carcinoma and adenocarcinoma tissues.

**Conclusions:**

Our data suggest the aberrant expression of *TNNT2* in lung cancer and its prevalence increases with pathological severity.

## Background

Lung cancer is the most common cancer worldwide, accounting for the 2.21 million new cases, and 1.80 million deaths in 2020 (1). It is characterized into 2 histologic groups: 15% of small cell carcinoma, the common form of neuroendocrine tumor, and 85% of non-small cell lung carcinoma in all lung cancers. Non-small cell lung carcinoma is subclassified as squamous cell lung carcinoma, adenocarcinoma, and large cell lung carcinoma (2, 3). Comprehensive molecular profiles have shown the high rates of somatic mutations and genetic alteration (3), and over-expression of genes not considered oncogenes (4–6) in lung cancer. Establishment of useful markers is necessary to accurately classify early- and advanced- stage disease (7).

Cardiac troponin-T is a ~37 kDa protein that in human is encoded by the *TNNT2* gene (8). It is a part of the tropomyosin-binding complex together with troponin-C, and troponin-I, and is located on the thin filament of striated muscles of heart (9, 10). Cardiac troponin-T concentration in the serum is routinely measured in diagnosing acute coronary syndrome (11), and is also detected in cancer patients with myocardial injury following the anti-cancer therapy (12–15). Anti-cancer drugs (e.g., anthracycline, trastuzumab) can damage myocardial cells, meanwhile pro-inflammatory cytokines produced from cancer cells themselves may injure the myocardial tissues (13). We reported the case who elevated cardiac troponin-T level in the serum in neuroendocrine ethmoid carcinoma receiving the immune checkpoint inhibitor. In this case, myocardial injury was not manifested, and we found the immunoreactivity of *TNNT2* in metastatic tumor cells (16). These implies that cancer cells have potential to express *TNNT2*. This study aimed to evaluate the frequency of *TNNT2* expression in neoplastic tissues.

## Methods

This study was approved by the Research Ethics Committee of Faculty of Medicine, University of Miyazaki (0-0503, 0-0706-1) and conformed to the principles outlined in the World Medical Association Declaration of Helsinki (17).

### Study Population

#### Experiment 1

We retrospectively enrolled 68 patients with a series of lung cancer at Miyazaki University Hospital, Japan between January 1, 2017, and March 31, 2019. We randomly chose paraffin-embedded blocks based on the records of clinical and pathological findings. We used paraffin-embedded blocks of 40 from operation, and 28 through biopsy. We obtained patients' information, such as age, sex, staging according to the tumor–node–metastasis (TNM) system, anti-cancer therapy (chemotherapy, radiation, and immune checkpoint inhibitor), and histology in the medical records. We divided into two groups; early-stage disease included patients performed surgical resection (Stage 0–I), and advance-stage included patients with surgical resection (Stage II), and eligible for chemotherapy and immune checkpoint inhibitors, and biopsy under thoracotomy (Stage III–IV and recurrence). We applied Opt-out method to obtain the consent on this study (Research Ethics Committee permission number: 0-0503).

### Histology and Immunohistochemistry

Tissue sections of 3 μm in thickness were autoclaved at 120°C for 20 min in 10 mmol/L citrate buffer (pH 6.0). The slide sections were immersed in 3% hydroxyl peroxide for 20 min to block the endogenous peroxidase, and thereafter incubated with Protein Block Serum–Free Ready–To–Use (Dako Cytomation) for 10 min to reduce the nonspecific background. They were incubated with the primary monoclonal antibody against *TNNT2* protein (18) (Clone 13–11, 1 μg/ml, catalog number #MA5-12960, Invitrogen) at 4°C overnight. The slides were incubated with EnVision^+^ System–horse radish peroxidase labeled polymer (Dakocytomation) for 30 min, visualized with 0.05% 3,3'–diaminobenzidine containing hydrogen peroxide, and counterstained with hematoxylin. We confirmed the specificity of antibody by Western blots (Supplementary Figure 1). We used a slide section of human heart as a positive control staining and carried out negative control staining by omitting the first antibody (data not shown). We used Allred score to combine the percentage of positive cells and the intensity of the reaction product in lung cancer (19). The two scores were added together for a final score. Scores of 0 and 2 were considered negative. Scores of 3 and 8 were considered positive (Supplementary Figure 2). In addition, resected non-mucinous adenocarcinomas were classified into 3 gradings (well-differentiated: lepidic-predominant with no or <20% high-grade pattern; moderately differentiated with no or <20% high-grade pattern: acinar or papillary-predominant; poorly differentiated: any tumor with ≥20% high-grade pattern (solid, microcapillary, cribriform, or complex glandular pattern), meanwhile squamous cell carcinomas were classified into keratinizing and non-keratinizing ones, according to the WHO Classification of Tumors (5^th^ Edition. Thoracic Tumors. Edited by the WHO Classification of Tumors Editorial Board).

#### Experiment 2

Additionally, we collected lung cancer tissues prospectively from four patients during the rapid intraoperative pathological diagnosis. Written informed consent was obtained from each patient before operation (Research Ethics Committee permission number: 0-0706-1).

### Reverse Transcription-Polymerase Chain Reaction

Resected lung cancer tissues were immersed in RNAlater^®^ (Ambion) at 4°C overnight. The tissue was pulverized in Isogen (Nippon Gen), and total RNA was extracted by the RNeasy^®^ Mini Kit (QIAGEN, Hilden, Germany). One μg of total RNA was used to synthesize complementary DNA by RT^2^ First Strand Kit (QIAGEN). Gene expressions of *TNNT2* and glyceraldehyde-3-phosphate dehydrogenase (*GAPDH*) were analyzed using RT-PCR. The following oligonucleotide primers were designed; *TNNT2*: AATGAGTTGCAGGCGCTGAT (forward, 370–389), CCGCTCTGTCTTCTGGATGT (reverse, 632–651); *GAPDH*: GAAGGTGAAGGTCGGAGTCA (forward, 82–101); TCGCTCCTGGAAGATGGTGA (reverse, 297–316). The reaction was performed in 12 μl, containing 1.2 μl of 10×E× Taq Buffer (Takara Bio Inc; Shiga, Japan), 0.06 μl TakaRa Ex Taq (5 units/μl), 0.96 μl dNTP mixture (dATP, dCTP, dGTP, and dTTP: 0.2 mmol/L of each), 0.1 μl of both forward and reverse primers (0.83 μmol/L), and 2.0 μl cDNA template. The amplification protocol was 94°C for 2 min, then 30 cycles of 94°C for 30 s, 61°C for 30 s and 72°C for 30 s, and finally 72°C for 10 min. A 10 μl aliquot of PCR products was separated on 2% agarose gel with ethidium bromide. The resulting PCR products were 282 bp for *TNNT2*, and 235 bp for *GAPDH*, respectively. Complementary DNA of human heart was used for positive control.

## Statistical Analysis

Data were analyzed using GraphPad Prism version 8.43 for Windows, GraphPad Software, La Jolla California, USA. Data were expressed as means ± standard deviation. Chi-squared test was performed with Excel 2010. *P* < 0.05 considered to be statistically significant.

## Results

### Baseline Characteristics

The baseline characteristics of our study population are displayed in Table 1. Cardiovascular disease includes 28, hypertension; 4, cerebral infarction; 1, coronary artery disease; 2, aortic dissection; 1, aortic aneurysm; 2, valvular disease; 2, left ventricular hypertrophy; 3, cerebral artery aneurysm; 2, pulmonary hypertension; 1, arteriosclerosis obliterans.

**Table 1:** Patients' characteristics.

**Number**	**68**
Age (Mean ± SD)	68 ± 10
Male gender, *n* (%)	42 (62%)
**Tumor stage**	
Stage 0, *n* (%)	5 (7%)
Stage I, *n* (%)	28 (41%)
Stage II, *n* (%)	6 (9%)
Stage III, *n* (%)	5 (7%)
Stage IV, *n* (%)	16 (24%)
Recurrence, *n* (%)	8 (12%)
Chemotherapy	24 (35%)
Radiation	9 (13%)
Immune checkpoint inhibitor	27 (40%)
Cardiovascular disease	41 (60%)
**Histologic type**	
Squamous cell carcinoma, *n* (%)	13 (19%)
Adenocarcinoma, *n* (%)	50 (74%)
Neuroendocrine cell carcinoma, *n* (%)	4 (6%)
Large cell carcinoma, *n* (%)	1 (1%)
*TNNT2* positive staining, *n* (%)	25 (37%)

*SD, standard deviation; n, number of patients; TNNT2, cardiac troponin-T*.

### Immunoreactivity of *TNNT2* Protein in Lung Cancer Tissues

**Figure 1:**
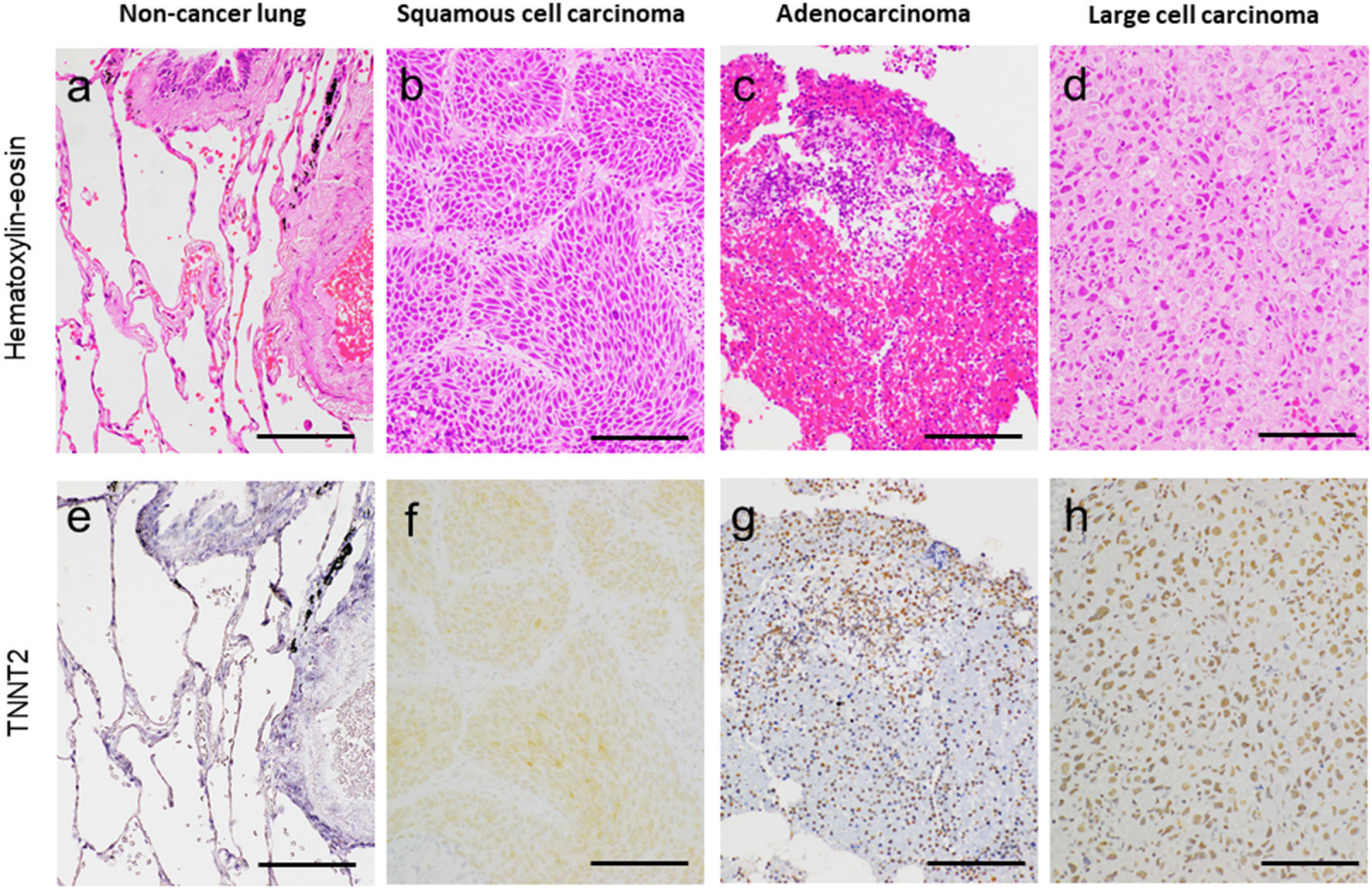
Representative pictures for hematoxylin–eosin **(a–d)** and cardiac troponin-T (*TNNT2*) **(e–h)** in non-cancer lung adjacent to cancer, lung squamous cell carcinoma, adenocarcinoma, and large cell carcinoma. Bar, 100 μm.

**Figure 2:**
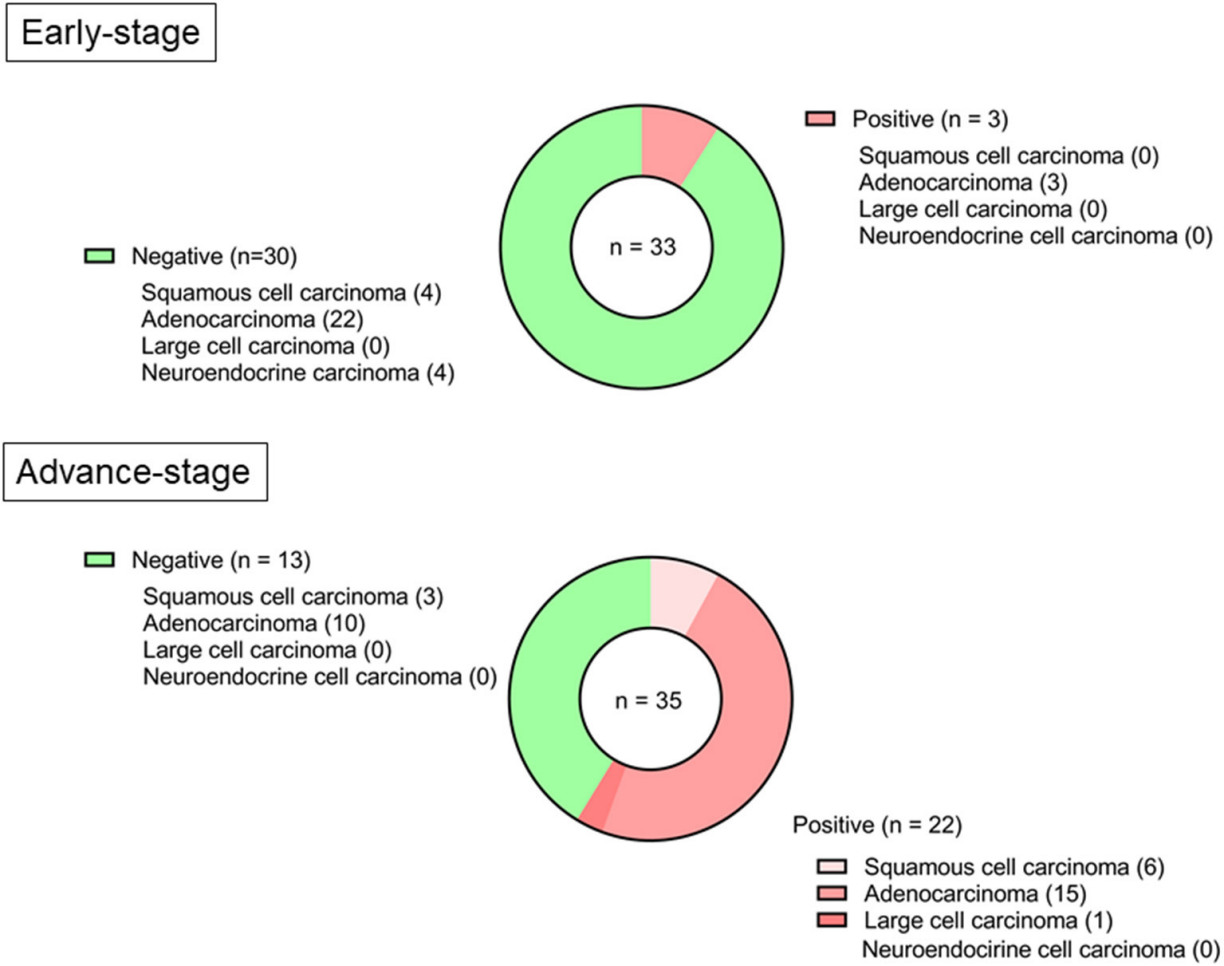
Frequency for *TNNT2* immunoreactive staining in specimens with early-stage lung cancer (Stage 0–I) and advance-stage lung cancer (Stage II–IV and recurrence).

**Figure 3:**
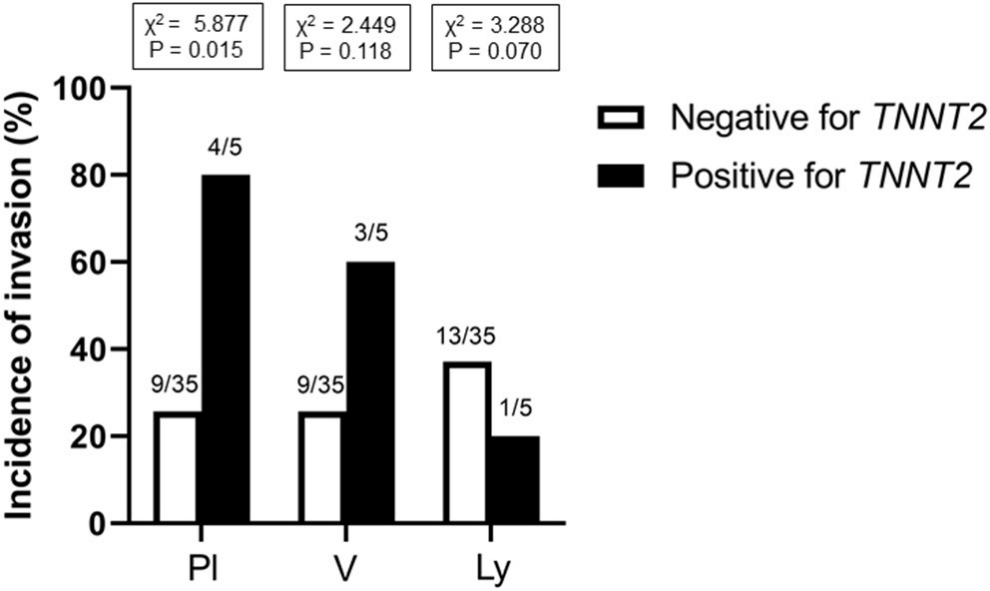
Incidence of *TNNT2* immunoreactive staining with pleural, vessel and lymphatic invasion in specimens performed surgical resection. Pl, pleura; V, blood vessel; Ly, lymphatic vessel.

Figure 1 shows the representative pictures for *TNNT2* in squamous cell carcinoma, adenocarcinoma, and large cell carcinoma. Immunoreactivity for *TNNT2* protein was localized in the cytoplasm and nucleus of cancer cells. Figure 2 summarizes the numbers of positive or negative staining for *TNNT2* in lung cancer specimens. In early-stage lung cancer patients (*n* = 33), *TNNT2* immunoreactivity was positive in three of adenocarcinoma, and negative in 30 (4, squamous cell carcinoma; 22, adenocarcinoma; 4, neuroendocrine cell carcinoma). In advance-stage lung cancer (*n* = 35), 22 specimens showed positive for *TNNT2* (6, squamous cell carcinoma; 15, adenocarcinoma; 1, large cell carcinoma), and negative in 13 (3, squamous cell carcinoma; 10, adenocarcinoma). In specimens performed surgical resection, frequency of positive staining for *TNNT2* increased with pleural (χ^2^ = 5.877, *P* = 0.015) and vascular (χ^2^ = 2.449, *P* = 0.118) invasions, while it decreased with lymphatic invasion (χ^2^ = 3.288, *P* = 0.070) (Figure 3).

### Relation Between *TNNT2* Expression and Histological Grading in Non-mucinous Lung Adenocarcinoma and Squamous Cell Carcinomas

*TNNT2*-positive non-mucinous adenocarcinomas exhibited the following histological grading: 0, well-differentiated; 8, moderately differentiated; 5, poorly differentiated. *TNNT2*-negative ones showed 7, well-differentiated; 11, moderately differentiated; 5, poorly differentiated. In addition, *TNNT2* was positively stained in 0, keratinizing; 1, non-keratinizing squamous cell carcinomas, meanwhile *TNNT2* protein was negative in 5, keratinizing; 1, non-keratinizing ones.

### *TNNT2* Staining at Primary Lesion and Its Recurrence/Metastasis

We stained specimens at primary lesion, and recurrent/metastatic sites in 11 patients with *TNNT2* antibody. Four of them revealed negative stain at both primary lesion and metastatic sites, and 3 cases showed negative stain at primary lesion and positive stain at recurrence. Other 4 cases showed positive staining at both primary lesion and metastatic/recurrent site.

### *TNNT2* Staining in Patients Received Immune Checkpoint Inhibitors

Immunoreactivity for *TNNT2* protein was positive in 5, squamous cell carcinoma; 16, adenocarcinoma; 1, large cell carcinoma in 27 patients who received immune checkpoint inhibitors. One patient with recurrent adenocarcinoma developed biopsy-proven myocarditis following the immune checkpoint inhibitor, pembrolizumab (20).

### Gene Expression of *TNNT2* in Lung Cancer

**Figure 4:**
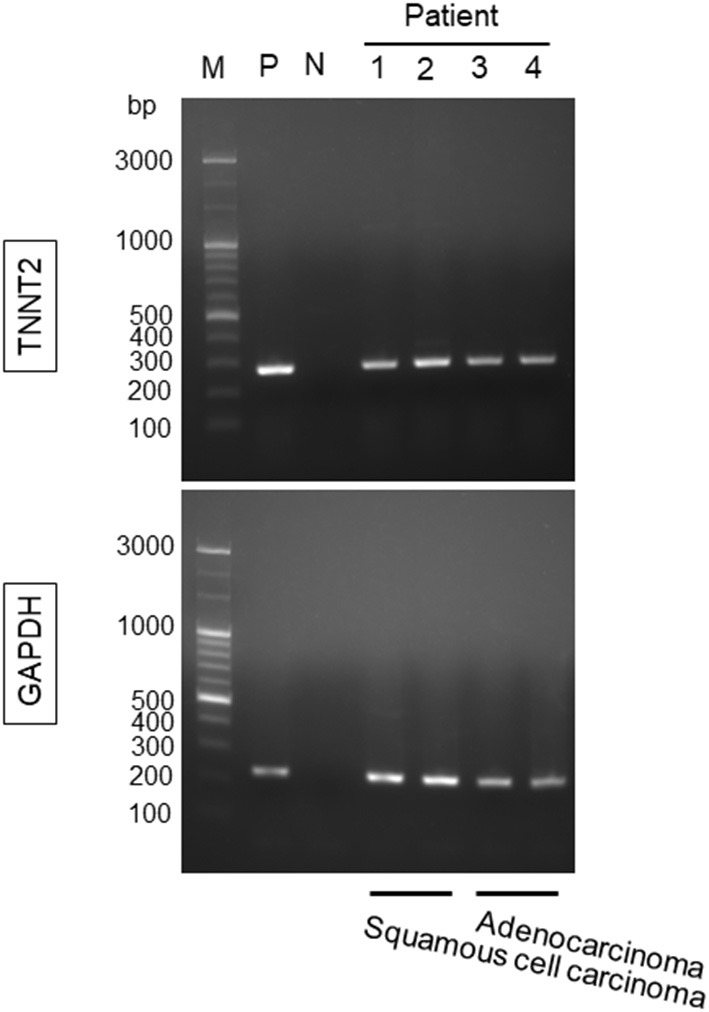
Gene expression for *TNNT2* in lung squamous cell carcinoma and adenocarcinoma. Lung cancer tissues were obtained from four patients (2, squamous cell carcinoma; 2, adenocarcinoma) during the rapid intraoperative pathological diagnosis. M, marker; P, positive control (human heart); N, negative control; 1 and 2, lung squamous cell carcinoma; 3 and 4, lung adenocarcinoma. GAPDH was used as a reference gene.

Lung cancer tissues obtained from four patients (2, squamous cell carcinoma, and 2, adenocarcinoma) demonstrated the expression of *TNNT2* mRNA (Figure 4).

## Discussion

The goal of this study was to evaluate the cardiac troponin-T (*TNNT2*) expression in neoplastic tissue. This study reports that *TNNT2* is present in lung cancer, irrespective of histologic types, and its prevalence increases with pathological severity.

Our data support studies that non-small cell lung cancers expressed cardiac troponin-I (*TNNI3*) (6), and colorectal cancers expressed *TNNT2* (21). This study advances our understanding that an aberrant expression of *TNNT2* in lung cancer cells, irrespective of histologic type, and in association with pathological severity. We confirmed that gene expression of *TNNT2* was detected in two major histologic types of lung cancer tissues. The functional roles of *TNNT2* in cancer cells remain unknown. In this study, *TNNT2* immunoreactivity was not observed in well-differentiated adenocarcinomas and keratinizing squamous cell carcinomas, and it seems likely that metastatic and/or recurrent neoplastic cells exhibit high prevalence of *TNNT2* immunoreactivity. The immunoreactivity for *TNNT2* was localized in the nucleus, and this suggests that *TNNT2* functions as a transcriptional factor in cancer cells (10), and might exert pro-metastatic and tumorigenic activity (22). In support of these hypotheses, Jing et al. (21) studied that colorectal cancer cell lines transfected with *TNNT2* induced the expressions of epidermal growth factor receptor and fatty acid synthase (23), important factors for tumor growth and metastasis. The COSMIC databases (http://cancer.sanger.ac.uk/cosmic) and the Human Protein Atlas database (https://www.proteinatlas.org) reveal that *TNTT2* is widely distributed in various types of cancer cells with amplification, deletion, or mutation of the gene, and its protein level is detected in colorectal cancer and papillary adenocarcinoma of thyroid. We are currently working the overexpression of *TNNT2* in cancer cell lines on cellular kinetics and motility. We found that pleural and vascular invasion were frequently seen in the *TNNT2*-positive cases. On the other hand, inverse correlation was seen on the lymphatic invasion. *TNNT2* protein is extrapolated to interact with NOTCH3 from the protein-protein interaction database (https://www.uniprot.org). NOTCH signaling stimulates to tumor angiogenesis (24), while it inhibits the lymphatic vessel sprouting induced by vascular endothelial growth factor (25). Further investigation is necessary to clarify this concern. Next, immune checkpoint inhibitors exert anti-tumor immunity, but also induce immune-related adverse effects in myocardium (14, 26). Immune checkpoint inhibitor was administered to 22 patients (5, squamous cell carcinoma; 16, adenocarcinoma; 1, large cell carcinoma) positive for *TNNT2* protein in biopsy specimens. During the follow-up, only one patient who presented positive staining for *TNNT2* protein developed fatal myocarditis after receiving the immune checkpoint inhibitor (20). It remains to be determined whether the cross-reactivity of *TNNT2* protein between clonal expansion of a T-cell population activated by tumor cells and on cardiomyocytes is one of the putative mechanisms of myocardial toxicity in this study (27, 28).

*TNNT2* is exclusively expressed in cardiac muscles, and it releases into the circulation by reflecting the extent of myocardial injury in acute coronary syndrome, myocarditis, cardiomyopathy, and anti-cancer therapy-induced cardiotoxicity (11, 14). With the progression of technology, high-sensitive assay detects the elevated cardiac troponin-T in patients with extra-cardiac disorders, including sepsis, stroke, pulmonary embolism, chronic renal failure, and skeletal myopathy (29–31). Pavo et al. (13) reported that cardiac troponin-T concentration increased in the serum with cancer patients without cardiotoxic anti-cancer therapy, and it is related to all-cause mortality. Finke et al. (32) recently reported that high levels of circulating cardiac troponin-T before starting chemotherapy predicted all-cause mortality after adjusting to glomerular filtration rate, age, gender, hypertension, diabetes, and adiposity, in which 86.7% (806 of 930) patients having a left ventricular ejection fraction > 50%. Prior cardiovascular disease, co-morbidity, hemodynamic instability, and pro-inflammatory cytokines produced from cancer cells damaged myocardial cells might affect the circulating cardiac troponin-T level (13, 14, 33, 34). Assessment of troponin-T helps to identify patients with cardiotoxicity during cancer therapy (35), and preventative therapy with β-blocker and angiotensin converting enzyme inhibitor/angiotensin II receptor blocker were associated with less cardiac troponin-T elevation in cancer patients (36). Although the circulating troponin-T levels in the peripheral blood were not evaluated at the time of tumor resection/biopsy, this study suggests a considerable attention may need to interpretate the cardiac troponin-T levels in the serum of cancer patients; circulating troponin-T from the tumor tissue may give false diagnosis of myocardial injury (16). However, it is noted that serum cardiac troponin-T level can be elevated with pre-existing/subclinical cardiac or noncardiac disorders without manifested cardiac dysfunction, meanwhile apparent normal cardiac function with elevated cardiac troponin-T cannot deny the presence of latent myocardial injury (34, 37). We observed *TNNT2* positive staining regardless of presence (+) or absence (-) of cardiovascular comorbidities (CVM): *TNNT2(-)/*CVM(-), 13; *TNNT2(-)/*CVM(+), 30; *TNNT2(*+*)/*CVM(-), 14; *TNNT2(*+*)/*CVM(+), 11 in 68 cancer patients. Further studies are necessary to investigate whether the positive immunoreactivity of *TNNT2* in tissues influences the circulating levels.

## Limitations

One potential limitation of this study is the unselective enrollment of patients, including the pathology of lung cancer. We included 4 cases of neuroendocrine cell carcinoma, and expected high incidence of positive immunostaining for *TNNT2* protein, based on our experience (16). However, advanced cases of neuroendocrine carcinoma were not listed in this study. This may be a potential reason why *TNNT2* expression is not observed in neuroendocrine carcinomas. Second, sample size was small, and selection bias might have affected the prevalence of positive staining for *TNNT2* under the variation in sample type and preparation. Third, only 37% of population exhibited the positive *TNNT2* staining, and we could not determine the prognostic value in lung cancer specimens with many other confounding variables.

## Conclusion

This study provides new insights into the *TNNT2* expression in lung cancer tissues. Its prevalence increased with the pathological severity, suggesting its relation to cancer progression. Biological function of *TNNT2* in lung cancer needs to be determined in the future study.

## Data Availability Statement

The original contributions presented in the study are included in the article/Supplementary Materials, further inquiries can be directed to the corresponding author.

## Ethics Statement

The study was approved by the Research Ethics Committee of Faculty of Medicine, University of Miyazaki. The patients/participants provided their written informed consent to participate in this study.

## Author Contributions

TT and YS: writing—original draft preparation. KN, NY, and RI: writing—review and editing. MT, YS, and HT: sample collection. TT, YS, and KH: histological analysis. MO and AK: molecular analysis. YA and KK: supervision. All authors critically revised the manuscript and agreed with the final version.

## Funding

This study was supported by grants-in-aid for Scientific Research (C) (19K08521 to TT) from the Japan Society for the Promotion of Science and Clinical Research from Miyazaki University Hospital.

## Conflict of Interest

The authors declare that the research was conducted in the absence of any commercial or financial relationships that could be construed as a potential conflict of interest.

## Publisher's Note

All claims expressed in this article are solely those of the authors and do not necessarily represent those of their affiliated organizations, or those of the publisher, the editors and the reviewers. Any product that may be evaluated in this article, or claim that may be made by its manufacturer, is not guaranteed or endorsed by the publisher.
