# Supplementary figures and images for: Aberrant Expression of Cardiac Troponin-T in Lung Cancer Tissues in Association With Pathological Severity
Source: Front Cardiovasc Med. 2022 Apr 11;9:833649. doi: 10.3389/fcvm.2022.833649 (PMC9037982; doi:10.3389/fcvm.2022.833649)

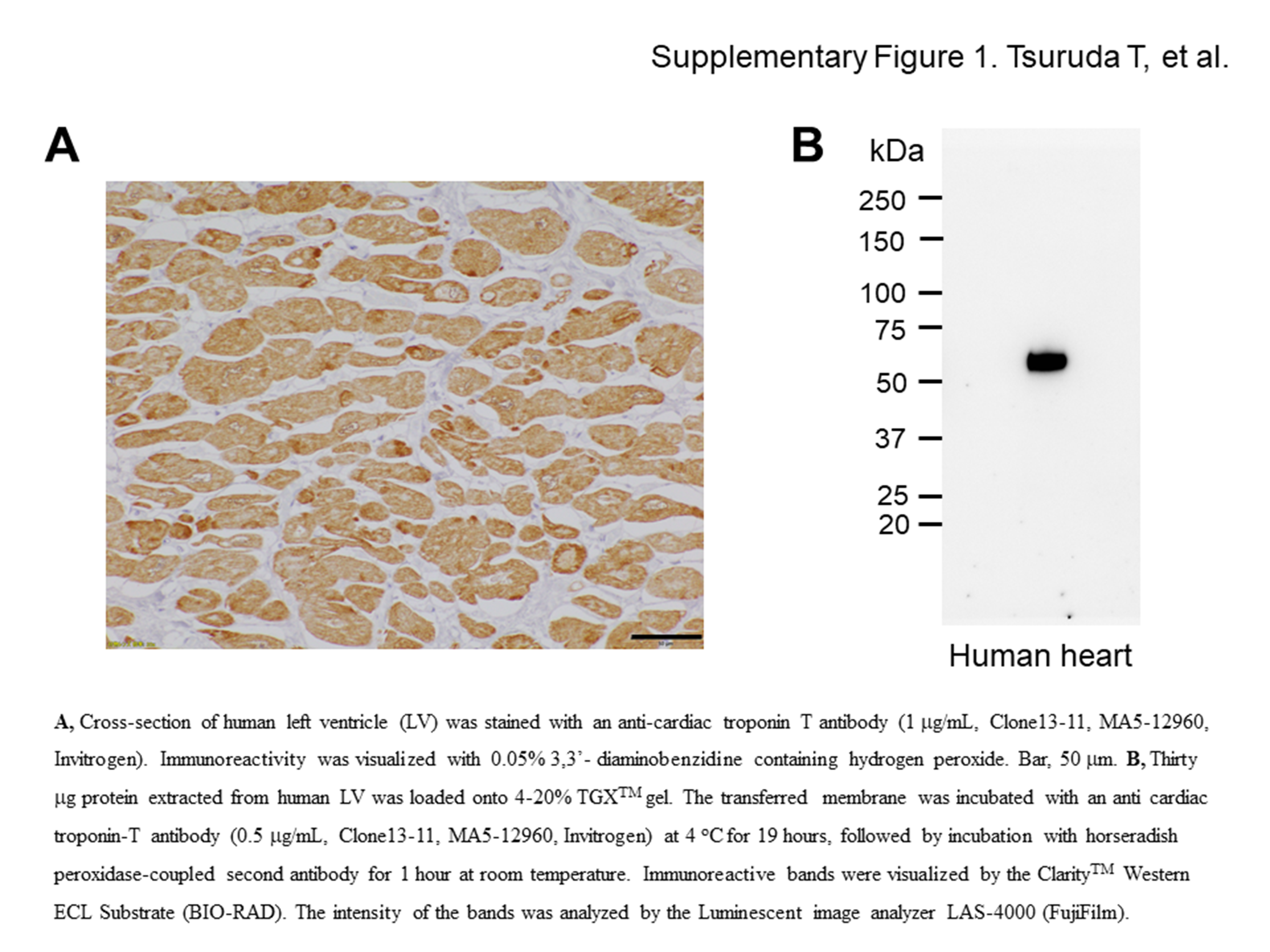

Supplement: Supplementary file 1 [file Image_1.TIF]

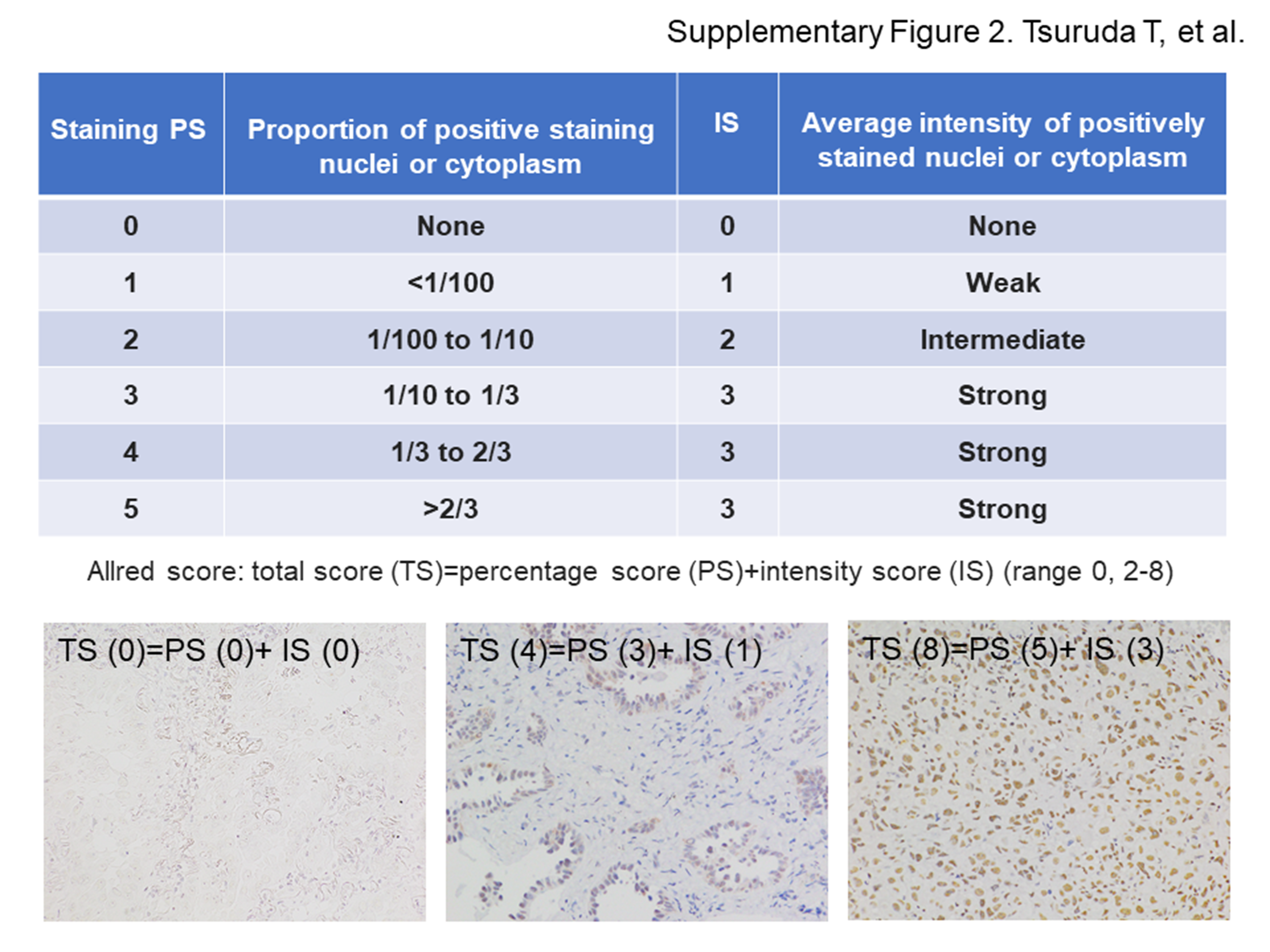

Supplement: Supplementary file 2 [file Image_2.TIF]
